# Anaesthesia Management for Giant Intraabdominal Tumours: A Case Series Study

**DOI:** 10.3390/jcm13051321

**Published:** 2024-02-26

**Authors:** Olga Grăjdieru, Cristina Petrișor, Constantin Bodolea, Ciprian Tomuleasa, Cătălin Constantinescu

**Affiliations:** 1 Department of Anaesthesia and Intensive Care, Iuliu Hatieganu University of Medicine and Pharmacy, 400349 Cluj-Napoca, Romania; olgadoinag@yahoo.com (O.G.); petrisor.cristina@umfcluj.ro (C.P.); bodolea.constantin@umfcluj.ro (C.B.); 2 Department of Hematology, Medfuture Research Center for Advanced Medicine, Iuliu Hatieganu University of Medicine and Pharmacy, 400337 Cluj-Napoca, Romania; ciprian.tomuleasa@gmail.com

**Keywords:** giant tumours, giant ovarian tumours, general anaesthesia, intubation under conscious sedation, awake intubation, spontaneous respiration, slow aspiration, supine hypotension, re-expansion pulmonary edema

## Abstract

Background: Due to a lack of randomised controlled trials and guidelines, and only case reports being available in the literature, there is no consensus on how to approach anaesthetic management in patients with giant intraabdominal tumours. Methods: This study aimed to evaluate the literature and explore the current status of evidence, by undertaking an observational research design with a descriptive account of characteristics observed in a case series referring to patients with giant intraabdominal tumours who underwent anaesthesia. Results: Twenty patients diagnosed with giant intraabdominal tumours were included in the study, most of them women, with the overall pathology being ovarian-related and sarcomas. Most of the patients were unable to lie supine and assumed a lateral decubitus position. Pulmonary function tests, chest X-rays, and thoracoabdominal CT were the most often performed preoperative evaluation methods, with the overall findings that there was no atelectasis or pleural effusion present, but there was bilateral diaphragm elevation. The removal of the intraabdominal tumour was performed under general anaesthesia in all cases. Awake fiberoptic intubation or awake videolaryngoscopy was performed in five cases, while the rest were performed with general anaesthesia with rapid sequence induction. Only one patient was ventilated with pressure support ventilation while maintaining spontaneous ventilation, while the rest were ventilated with controlled ventilation. Hypoxemia was the most reported respiratory complication during surgery. In more than 50% of cases, there was hypotension present during surgery, especially after the induction of anaesthesia and after tumour removal, which required vasopressor support. Most cases involved blood loss with subsequent transfusion requirements. The removal of the tumor requires prolonged surgical and anaesthesia times. Fluid drainage from cystic tumour ranged from 15.7 L to 107 L, with a fluid extraction rate of 0.5–2.5 L/min, and there was no re-expansion pulmonary oedema reported. Following surgery, all the patients required intensive care unit admission. One patient died during hospitalization. Conclusions: This study contributes to the creation of a certain standard of care when dealing with patients presenting with giant intraabdominal tumour. More research is needed to define the proper way to administer anaesthesia and create practice guidelines.

## 1. Background

Patients presenting with intraabdominal masses or tumours, especially giant ones, are considered high-risk surgical and anaesthesia patients. Even though the incidence should be decreasing due to access to medical facilities, early detection, and improved education among the population, there are still patients who, unfortunately, tend to present to the hospital at later stages of the disease, when the primary condition has started causing severe systemic signs and symptoms. The main reasons for this could be the lack of medical education in different geographic areas, psychological factors, social deprivation, and a lack of understanding of the nature of their disease. Unfortunately, giant intraabdominal tumours have a wide range of origins, but the ones that can grow quite huge are represented by ovarian tumours, sarcomas, and pseudomyxomas peritonei [1,2,3,4]. There is a dire need to develop guidelines to improve the recognition of systemic complications and appropriate surgical and anaesthesia management when approaching giant intraabdominal tumour. Preoperative assessment, optimization, and the careful planning of anaesthesia management are mandatory for reducing perioperative complications [5,6].

To assess the suitability of surgery and optimise premorbid status, a multidisciplinary approach is essential for careful preoperative evaluation. Physicians from different departments, like general surgery, gynecology, oncology, radiology, pathology, gastroenterology, anaesthesia, and intensive care, should gather before surgery to discuss the overall strategies for the case. They should consider logistical issues, the type of tumour and its impact on physiological parameters, the surgical approach, the anaesthetic plan, surgery and anaesthesia-related complications, and postoperative management in intensive care facilities.

Large intraabdominal tumours are responsible for alterations in normal physiologic parameters. Due to the increased intraabdominal pressure and compression by the tumor, they can cause respiratory, hemodynamic, and gastrointestinal symptoms [7,8]. Furthermore, changes in intrathoracic pressure due to patient positioning and intraoperative intermittent positive pressure ventilation can lead to hemodynamic collapse [9]. Of note, haemorrhage is a common complication during the surgical removal of the mass, which anaesthesiologists should anticipate and prepare for.

**Figure 1:**
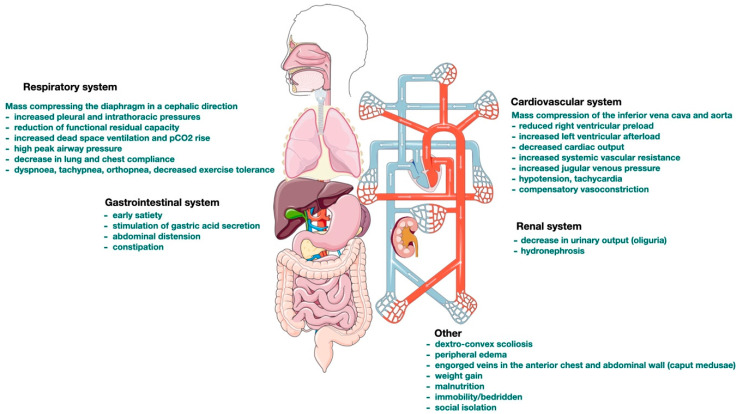
General complications of giant intraabdominal tumours.

By splitting the anaesthetic phases into before and after tumour removal, several important features are worth mentioning. Before tumour removal, there are respiratory, hemodynamic, renal, and gastrointestinal tract complications (Figure 1). After tumour removal, there is a rapid decrease in intrathoracic pressure, hemodynamic collapse, and the possibility of the development of re-expansion pulmonary oedema (RPE) [10].

Surgery-related problems are focused on the possibility of preoperative drainage of cystic tumour, postoperative intestinal distension, and postoperative analgesia. The debate regarding the pre- or intraoperative drainage of massive cystic tumour remains active. Preoperative drainage could offer no advantage even if it is discussed on a case-by-case basis, and the question of whether it should be done before surgery remains unanswered [11]. The spread of cancer, intraabdominal infections, bleeding, and adhesions are potential complications of drainage, and thus could make this procedure inadvisable. Intraoperative drainage could be favorable as long as it is done gradually to allow the anesthesiologists to fill the empty vascular bed to treat the systemic hypotension that follows the removal of the mass from the abdominal compartment.

The rationale of this study is to propose a standard of conduct for anaesthesia and a standard of care for patients presenting with giant intraabdominal tumours.

## 2. Material and Methods

This study aimed to evaluate the literature and explore the current status of evidence by performing an observational research design with a descriptive account of characteristics observed in a case series referring to patients with giant intraabdominal tumours who underwent surgery under general anaesthesia. 

We have searched the literature for English-language case reports from 1997 to 2022 describing the approach to giant intraabdominal tumours with an emphasis on the management of anaesthesia. We included twenty case reports from the literature (Table 1). The inclusion criteria were patients who were 18 years of age or older, had been diagnosed with a giant intraabdominal tumour, and had to undergo surgery under general anaesthesia. The exclusion criteria were paediatric patients and a lack of information regarding anaesthesia management. Even though we found there were a lot of reported cases, they did not meet all the inclusion criteria, so they were excluded. 

The main outcomes of interest were mortality, and duration of hospital and intensive care unit (ICU) stay. Secondary outcomes were the weight of the tumour, the duration of surgery and anaesthesia, the type of induction of anaesthesia (awake or rapid sequence induction), respiratory and haemodynamic complications during surgery, total blood loss with blood requirement, fluid extraction rate in cystic tumours and total fluid drainage. Overall knowledge in this field is scarce, and there is a lack of systematic randomised trials. Also, it is worth mentioning that there are some variables for which there is missing information among the reported cases.

Statistical analysis and graphics were performed using the statistical software R Studio (version 2023.03.0+386).

## 3. Results

**Figure 2:**
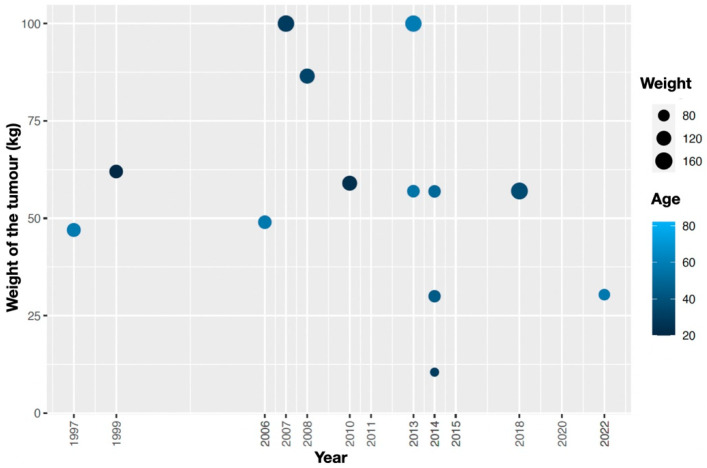
The year of the reported case is listed against the weight, age, and reported weight (kg) of the tumour.

Out of the twenty patients included in the study, there were seventeen females and three males, with the overall pathology being ovarian-related and sarcomas. Data regarding the year of the reported case, age, weight, BMI, IBW, and weight of the tumour are available in Figure 2. The patients’ mean age ± SD was 46 ± 16.6. 

Most of the patients were unable to lie supine and assumed a lateral decubitus position with normal peripheral oxygen saturation (SpO2) in atmospheric air. 

**Figure 3:**
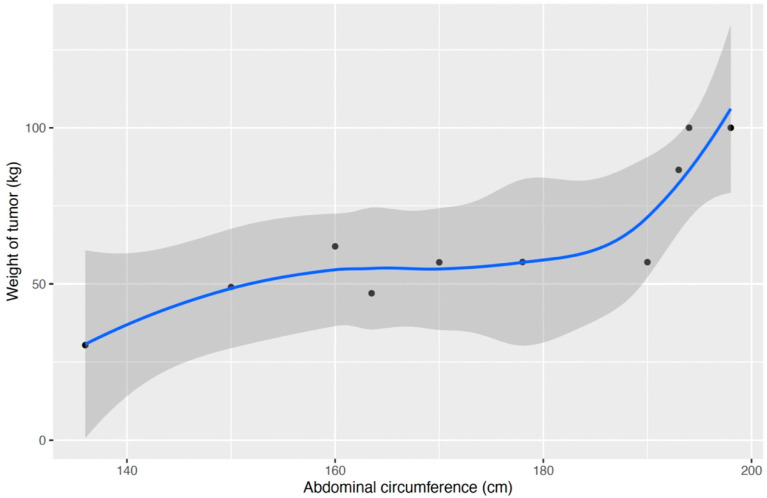
Abdominal circumference (cm) against weight of the tumor (kg). There is a strong positive linear relationship between these two variables (correlation coefficient of 0.85).

Pulmonary function tests, chest X-rays, and thoracoabdominal CT were the most often performed preoperative evaluation methods. Preoperative pulmonary function tests revealed either normal or restrictive impairment. The chest X-ray revealed elevation of the bilateral diaphragm. Thoracoabdominal CT was performed in 75% of cases, and the overall findings were that there was atelectasis or pleural effusion present. The rest of the patients could not undergo the scanning due to the size of the tumour (Figure 3). 

Regarding surgery duration, a mean of 345 min was calculated from the overall reported values, and the mean of anaesthesia duration was 487 min. 

The removal of the abdominal tumour was performed under general anaesthesia in all the cases. The patient was positioned in a semirecumbent or left lateral decubitus position before induction of anaesthesia. Awake fiberoptic intubation or awake videolaryngoscopy was performed in five cases; the rest were performed with general anaesthesia with rapid sequence induction. After the induction of anaesthesia, only one patient was ventilated with pressure support ventilation while maintaining spontaneous ventilation, while the rest were ventilated using controlled ventilation. 

The most reported respiratory complication during surgery was hypoxemia. There was no re-expansion pulmonary oedema reported.

In more than 50% of cases, hypotension was reported during surgery, especially after the induction of anaesthesia and after tumour removal, which required vasopressor support. Invasive arterial blood pressure and central venous cannulation were performed in most of the cases.

Preoperative epidural insertion in the lumbar region for perioperative analgesia was performed in four cases, and there were no incidents reported.

**Figure 4:**
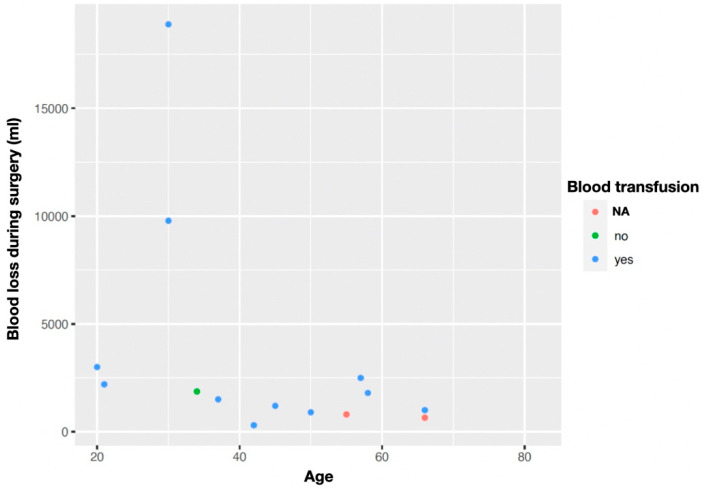
Blood loss during surgery (mL) and the need for transfusions.

Most cases involved blood loss with transfusion requirement (Figure 4).

Cystic tumours involved fluid drainage in the range of 15.7 L to 107 L, with a fluid extration rate of 0.5–2.5 L/min.

**Figure 5:**
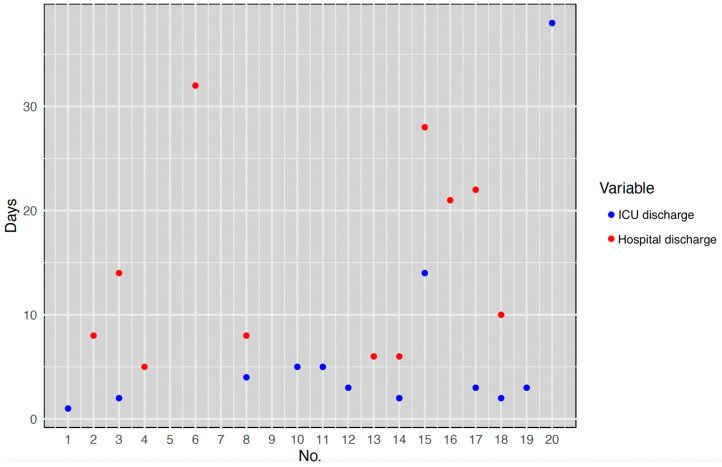
Day of ICU and hospital discharge.

All the patients required ICU management after surgery. The ICU and hospital duration of stay for each case are presented in Figure 5.

Only one patient died during hospitalisation.

## 4. Discussions

A search of the available literature found no integration of information reflecting the correct approach to anaesthesia for giant intraabdominal tumour.

From a historical perspective, giant intraabdominal tumours have been described in the literature starting in the twentieth century, at a time when anaesthesia was quite different. The first report describing the removal of a giant intraabdominal cyst weighing 148.6 kg was published in 1906 [31]. Other reports worth mentioning are the removal of an 83.4 kg tumour in 1954 [32], and a 137.5 kg tumour in 1994 [33], both of them being giant ovarian tumours.

Giant intraabdominal tumours are responsible for anatomical, physiological, and psychological changes, and represent a challenge for the surgeon as well as for the anesthetist. They can lead to increased intraabdominal hypertension or even abdominal compartment syndrome, thus the interval between the diagnosis and surgery should be as short as possible [34,35]. Although they do so rarely, they can cause thrombosis of the iliofemoral veins by direct compression [36]. Their growth in size over time leads to their stretching of the musculature of the abdominal wall beyond what is considered normal, and renders the patient confined to certain positions.

There are several main concerns and challenges in the management of these cases.

Due to increased intraabdominal pressure, these patients are considered to have a full stomach, so there is a risk of pulmonary aspiration during intubation. To rapidly secure the airway and avoid losing spontaneous respiration, awake fibre optic intubation or videolaryngoscopy should be performed [37,38]. Alternatively, rapid sequence induction with cricoid pressure could be a choice, although difficult ventilation and oxygenation may follow due to loss of spontaneous respiration [39]. In our study, awake fiberoptic intubation was performed in five cases, and only one patient was managed with pressure support ventilation during spontaneous respiration. This could be because anaesthesiologists are unfamiliar with awake intubation and prefer the comfort of classic anaesthesia.

Hiatal hernias can sometimes be present and pose additional challenges due to increased intrathoracic pressure with heart and lung compression [28]. Spontaneous breathing during intubation preserves the diaphragm’s contractile force to prevent further herniation of organs into the thorax, and avoids the increased intrathoracic pressure that would otherwise occur during mandatory ventilation [40]. The presence of a hiatal hernia should be sought before surgery. 

Furthermore, associated pneumonia can be a feature due to a poor ability to cough and the retention of secretions [25].

Another concern during the management of this kind of tumour is excessive bleeding, which can be experienced during adhesiolysis or due to the malignant type of tumour, which can be entirely adherent to the parietal peritoneum. This kind of bleeding can become life-threatening due to the difficulty of obtaining hemostasis with continuous oozing, and the development of intravascular disseminated coagulation due to massive transfusion [15]. Postoperative bleeding from the parietal wall is also described, which requires subsequent surgery and the transfusion of blood products [20,22]. Fourteen of the twenty reported cases had significant blood loss, and twelve required blood product transfusions. Therefore, due to the increased incidence of bleeding, blood-derived products should be readily available, along with rapid infusion devices.

Prolonged surgical times lead to the development of hypothermia due to the exposure of the peritoneal cavity and large fluid shifts [41]. Bleeding can further aggravate hypothermia due to the requirement for volume replacement and the transfusion of blood products. Even though there was no information about the patients’ temperatures during surgery in the reported cases, there were prolonged surgical times and bleeding; thus, passive and active warming techniques should be used. Respiratory and haemodynamic compromise occur as a result of the tumour’s intra-abdominal development and compression of organs. Large intraabdominal masses lead to elevation of the bilateral diaphragm, decreased lung compliance, and high airway pressures. 

Of note, further respiratory failure can occur after the administration of neuromuscular blocking drugs. The relaxation of the diaphragm together with increased intraabdominal pressure leads to decreased lung and thoracic compliance, which causes increased peak and plateau airway pressures. 

Thus, one way to avoid this complication is to maintain spontaneous respiration with pressure support ventilation until abdominal decompression is achieved, which could be followed by the initiation of controlled ventilation. The inspiratory pressure should be kept under 20 cm H_2_O [42], the airway plateau pressure under 30 cmH_2_O, and the driving pressure under 15 cm H_2_O [43]. The physicians should always keep in mind that there is a possibility that spontaneous respiration will become inefficient and positive pressure ventilation will eventually be required, along with the necessity of giving neuromuscular blocking drugs. At this point, the haemodynamic system could become partially compromised; therefore, this event should be anticipated and prepared for.

Regarding tumours that are cystic in nature and benign, the slow aspiration of fluid contents before surgical resection to reduce the tumour mass and maintaining spontaneous respiration are effective measures to prevent respiratory failure and the re-expansion of pulmonary oedema [12,44]. This cannot be enforced in cases where malignancy is suspected due to the risk of dissemination or the tumour’s solid nature. When choosing a slow drainage approach, daily for several days before surgery, it is important to acknowledge that there will be a longer hospital stay and restrictions on patient activity. After the fluid contents have been drained or the solid tumour has been resected, the patient can be positioned supine.

Supine hypotensive syndrome is well described in pregnancy [45]. Intraabdominal tumours can lead to supine hypotensive syndrome due to aortocaval compression, so this can be avoided by nursing the patient in the left lateral decubitus. A giant intraabdominal tumour can decrease venous return, decrease cardiac output, increase systemic vascular resistance, increase cardiac workload, and develop collateral circulation [46].

Moreover, sympathetic activity is decreased during general and epidural anaesthesia, which can lead to sympthomatic inferior vena cava syndrome in patients with giant intraabdominal tumours. These factors, when combined with inadequate preoperative volemic status and the initiation of positive pressure ventilation, can significantly reduce venous return [14].

Re-expansion pulmonary oedema (RPE) is considered a non-cardiogenic pulmonary edema. The pathophysiological mechanisms of RPE are a consequence of the duration and severity of collapsed lungs, loss of surfactant, increased pulmonary vascular permeability, changes in pulmonary artery pressure, or the presence of inflammatory cytokines (IL-8, leukotriene B4) [47]. Prolonged increased intraabdominal pressure causes a bilaterally elevated diaphragm with compression and collapse of the lung. RPE occurs as a consequence of the rapid expansion of the lungs after a long-term collapse. Lung collapse over a period of 3 or more days, or an evacuation volume of more than 2000 mL of fluid, can lead to a rapid onset of RPE within one hour of the re-expansion of the lung [10]. It is similar to the unilateral pulmonary oedema after the treatment of spontaneous pneumothorax [48].

Airway obstruction and the approach to lung re-expansion by recruitment manoeuvres can also cause RPE [49]. A lung protective ventilation strategy should be used during the entire invasive mechanical ventilation period [50]. Re-expansion of the atelectatic lung should occur gradually in order to prevent RPE and could be performed with spontaneous respiration [21]. Measuring tidal volume during spontaneous respiration can help in setting a similar tidal volume during controlled ventilation.

In cases of cystic tumours, the gradual aspiration of fluid in a slow manner at a rate of 0.5–1 L/min can be used to prevent re-expansion pulmonary oedema. Studies from the literature describe an extraction rate range of 22.2–33 L/h to successfully prevent RPE [11]. There was no RPE encountered among the reported cases, and cystic tumours involved fluid drainage in the range of 15.7 L to 107 L, with a fluid extraction rate of 0.5–2.5 L/min.

Currently, there is no consensus concerning the extraction rate, and there are no standard methods for preventing RPE.

The average ICU stay is 5 days, but physicians should expect a longer hospital stay.

Another concern is the issue of intestinal distention following surgery, which should be recognized. The causes of this complication are attributed to the diffusion of gases into the bowel lumen and increased sympathetic activity [51]. Prokinetic administration and the insertion of a nasogastric tube can help overcome the problem. There is only one case reported in which death occurred in one patient due to prolonged ileus with cecal perforation after the removal of a giant intraabdominal tumour [52].

In this case series, only one patient died due to severe blood loss from the parietal peritoneum followed by the development of intravascular disseminated coagulopathy [15].

Regarding the question of whether epidural insertion is safe in these cases, although it may have many advantages, the consequences that giant intraabdominal tumours have on the intraabdominal compartment and epidural space can lead to a few particularities that should be mentioned and taken into consideration. Due to increased intraabdominal pressure, the pressure in the epidural space increases, and the epidural venous plexus of Batson becomes engorged [53]. Together with technical difficulties, this leads to an increased risk of causing an epidural hematoma. Furthermore, haemodynamic instability can be aggravated by the sympatholytic effect of the local anaesthetics administered into the epidural space. Although, there are two cases in which an epidural was placed. In one patient the epidural was placed for the preoperative percutaneous drainage of a cyst. In the other case report, it was used for postoperative analgesia. There were no incidents reported, but none were used during surgery [12,27]. Thus far, the general recommendations suggest avoiding epidural anaesthesia for intraoperative, not postoperative, management of these patients.

Regarding the limitations of our study, the lack of overall randomized controlled studies that deal with patients with giant intraabdominal tumours undergoing surgery, and the availability of only case reports, impeded the creation of a more complex and complete database. As such, only observational research with a descriptive account of the characteristics observed could be performed. The small sample size, the heterogeneity of giant intraabdominal tumours, and the different backgrounds of the patients are responsible for the inability to make general assumptions. Incomplete and scarce information regarding the management of these patients contributes to the shortcomings and inability to create a joint standard of research and care. 

**Proposal for anaesthesia management in patients with giant intraabdominal tumours.**

**Preoperative assessment**

The examination should focus on obtaining a thorough medical history, weight, height, body mass index, abdominal circumference, measurement of intraabdominal pressure, nutrition status, blood tests, and tumour markers. In addition, further investigations such as an ECG, chest X-ray, and CT should follow to assess the suitability of surgery and planning of anaesthesia. Venous Doppler ultrasonography of the lower limbs should be performed to evaluate for the presence of deep venous thrombosis, as giant tumours might disrupt the blood flow.

Before continuing with surgery, the ABO and Rh blood groups, as well as the availability of packed red blood cells, fresh frozen plasma, and platelets, should be confirmed. Also, heating devices and compression stockings should be at hand.

**Intraoperative management**

It is imperative that the patient be adequately prepared, and usually this requires a prolonged preoperative preparation. The prophylaxis of aspiration pneumonia should be considered. Routine AAGBI/ASA mandatory monitoring should be in place, along with two large-bore intravenous peripheral catheters. Before the induction of anaesthesia, we should obtain an invasive arterial pressure measurement, insert a central venous catheter under ultrasound guidance, and insert a urinary catheter. Heamodynamic monitoring, including cardiac output (CO), pulse pressure variation (PPV), stroke volume variation (SVV), and continuous central venous pressure (CVP), could be measured with a FloTrac™/Vigileo™ system (Edwards Lifesciences, Tokyo, Japan), which can be used to assess fluid responsiveness during surgery [54,55]. Whenever possible, transthoracic or transesophageal Doppler ultrasound can also help manage haemodynamics during anaesthesia. Entropy and the Surgical Plethysmographic Index (SPI) can be used to monitor the depth of anaesthesia and the adequacy of analgesia, respectively.

The patient should be positioned in a semirecumbent position, or more often, in a left lateral decubitus. Awake intubation should be considered for securing the airway. Alternatively, rapid sequence induction with cricoid pressure should be performed. Administer topical 4% lidocaine from the posterior pharynx to the glottis while oxygenating with a mask at 10 L/min over 10 min, or with the use of a high-flow nasal cannula. A McKenzie system can be used to properly anaesthetize the airway [56]. Conscious sedation with target-controlled infusion (TCI) remifentanil can also be initiated. After securing the airway, propofol or sevoflurane can be used for the induction of anaesthesia. To maintain spontaneous respiration with pressure support ventilation, neuromuscular blocking drugs should be avoided. The loss of spontaneous respirations could cause ventilatory failure. Surgeons should be aware of the decreased venous return and hypotension that can occur due to increased intrathoracic pressure when initiating positive pressure ventilation. The maintenance of anaesthesia can be done with sevoflurane and remifentanil. Stable hemodynamic management should be sought, and vasopressor drugs should be readily available.

Surgery can be commenced in the semirecumbent position to prevent supine hypotensive syndrome and respiratory compromise. In cystic tumours, there should be a gradual aspiration of fluid at a rate of 0.5–1 L/min (to prevent RPE), which is usually followed by an improvement in pulmonary compliance and a decrease in blood pressure. The patient can be positioned supine after fluid aspiration or tumor resection. Due to the nature of the disease, besides the intraabdominal tumour, the resection of other organs could be required.

After decompression, there is a reduction in airway pressure, increased lung compliance, and improved gas exchange. An increase in stroke volume and cardiac output, and a decrease in SVV, are expected due to increased venous return to the heart. Analgesia can be achieved with infiltration of the incision site with ropivacaine (20 mL), 0.5–0.75%, before incision for an opioid-sparing effect and to allow the patient to maintain spontaneous respiration.

**Postoperative management**

Due to potential ongoing respiratory and haemodynamic compromise, blood loss, fluid shifts, or surgical complications, the patient needs to be admitted to an ICU in order to continuously monitor their vital signs. Further blood tests will be necessary, and blood transfusion may also be required.

Postoperative analgesia can be achieved with paracetamol, opioids, wound infiltration, or trunk blocks such as the transversus abdominis plane (TAP) block.

Due to relaxation of the abdominal and diaphragmatic musculature, delayed extubation is occasionally suggested, and can be accomplished in the ICU. An abdominal binder should be used during the recovery period.

**Practice points**

What is the optimal strategy for the management of anaesthesia for giant intraabdominal masses?

- Adequate patient preparation (multidisciplinary approach; pharmacologic; nutritional);
- Book an ICU bed before surgery;
- Make sure there are blood products available;
- Warm the patient before induction of anaesthesia;
- Optimal patient positioning—left lateral decubitus/semirecumbent position;
- If possible, perform intubation while awake;
- Maintain initial spontaneous respirations until abdominal decompression;
- Advanced haemodynamic monitoring and goal-directed fluid therapy;
- Gradual aspiration of the fluid (if cystic)—0.5–1 L/min;
- Beware of postoperative intestinal distention.

**Research agenda**

- Studies comparing the management of anaesthesia in giant intraabdominal tumours are needed to help define the optimal strategy and establish a standard of care for an individualised approach.
- Complex respiratory and haemodynamic monitoring (CO, SV, SVV, and SVR) before, during, and after surgery could reveal major differences. Imagistical studies with complex measurements could help in defining exactly what organs are compromised due to the compression of the tumour, and different positioning and surgical approaches could be suggested.
- Improving the quality of life.

## 5. Conclusions

A multidisciplinary approach is mandatory for the thorough perioperative management of giant intraabdominal tumours. Raising physician awareness of the systemic complications caused by these tumours, as well as the options for treatment and anaesthesia management, can lead to better outcomes. Proper patient positioning, awake intubation with spontaneous breathing preservation, targeted fluid therapy, and gradual aspiration of the cystic content to prevent the re-expansion pulmonary oedema are just a few of the details to be aware of.

Further clinical research in the form of randomized clinical trials is essential to understanding the perioperative complications of these diseases.

The current study highlights the need for a universal standard of care when managing patients with giant intraabdominal tumours.

## Figures and Tables

**Table 1:** Characteristics and pre-operative data of patients with giant intraabdominal tumours.

No./ Year/ Reference	Age	Sex	Weight	Height (m)	BMI	IBW (kg)	Abdominal Circumference (cm)	Pathology	Medical History	Weight of Tumour (kg)	Postoperative Weight (kg)
1 1997 [12]	58	female	107.6	1.5	48	43	163.5	mucinous cystadenoma of ovary	-	47	-
2 1999 [13]	21	female	103	1.53	44	46	160	mucinous borderline tumour of the intestinal type confined to the right ovary	malnutrition, dyspnea, amenorrhea	62	44
3 2006 [11]	57	female	102	1.65	37	57	150	mucinous cystadenoma of ovary	malnutrition, dyspnea	49	53
4 2006 [14]	42	male	-	-	-	-	-	autosomal dominant polycystic kidney disease	hypertension, CKD V	10.5	-
5 2007 [15]	30	female	148	1.63	56	55	198	mucinous cystadenoma of ovary	dyspnea	100	-
6 2008 [16]	34	female	125	1.5	56	43	193	serous cystadenoma of ovary	malnutrition, amenorrhea	86.5	39.3
7 2010 [17]	24	female	122	1.65	45	57	-	giant mucosal-serosal cystadenoma	dyspnea, congenital bilateral clubfoot	59	-
8 2011 [18]	45	female	-	-	-	-	140	serous cystadenomas of paraovarian or paratubal origin	-	-	-
9 2013 [19]	59	female	146	1.54	62	47	194	benign ovarian cyst	-	100	50
10 2013 [20]	55	female	90	-	-	-	190	mucinous cystadenoma of ovary	-	56.95	-
11 2014 [21]	30	female	57	1.61	22	53	-	mature teratoma without malignancy	ovarian germ cell tumor at age 12	10.5	-
12 2014 [22]	50	female	90	-	-	-	170	mucinous cystadenoma of ovary	-	56.9	32
13 2014 [23]	44	female	88	-	-	-	-	enteric type of multilocular mucinous ovarian cyst adenoma	-	30	57
14 2015 [24]	66	male	-	-	26	-	-	liposarcoma-retroperitoneal	-	4.5	-
15 2015 [25]	20	female	100	-	-	-	161	giant ovarian tumour	pneumonia, obstructive pyelonephrosis and hydroureter	-	-
16 2018 [26]	37	female	154.3	1.66	56	58	178	mucinous borderline tumour	asthma	57	85
17 2020 [27]	66	female	68	1.61	26	53	110	mucinous cystadenoma of ovary	-	-	-
18 2022 [28]	82	female	41.5	1.55	17	48	-	spindle cell tumor (desmoid type fibromatosis)	hypertension, valvular heart disease	-	-
19 2022 [29]	57	male	78	1.74	26	70	136	liposarcoma (retroperitoneal)	hypertension, smoker and COPD	30.4	-
20 2022 [30]	46	female	193.2	1.66	70	58	-	giant ovarian tumour	-	-	-

## Data Availability

The raw data supporting the conclusions of this article will be made available by writing to the corresponding author at constantinescu.catalin@umfcluj.ro, without undue reservation. Figure 1 and the graphical abstract were partly generated using Servier Medical Art, provided by Servier, licensed under a Creative Commons Attribution 3.0 unported license.

## Abbreviations

RPE	re-expansion pulmonary oedema
ICU	intensive care unit
CO	cardiac output
PPV	pulse pressure variation
SVV	stroke volume variation
SPI	Surgical Plethysmographic Index
TCI	target-controlled infusion
TAP	transversus abdominis plane
